# A Gap Between Policy and Practice: A Case Study on Maternal Mortality Reports, Kerman, Iran

**Published:** 2011

**Authors:** Farzaneh Zolala, Ali Akbar Haghdoost

**Affiliations:** 1 Kerman University of Medical Science, Faculty of Health, Haftbagh-Alavi Highway, Kerman, Iran; 2 Associate Professor of Epidemiology and Biostatistics, Department of Epidemiology and Biostatistics, School of Health, Kerman University of Medical Sciences, Kerman. Iran

**Keywords:** Maternal mortality, Policy, Practice, Prevention

## Abstract

**Objectives:**

Maternal mortality, a preventable tragedy, is an indicator of development, poverty and democracy. Therefore, prevention of maternal deaths is one of the main goals in many countries. Iran is one of the developing countries which aim to reduce maternal mortality through introduction of a new policy at national level. This study aimed to explore the extent to which this policy is practiced at peripheral levels.

**Methods:**

The data were collected through interviews with relevant people, observation, and review of the documents.

**Results:**

The results showed that there is a gap between policy and practice which can be explained by inadequate training programs, inadequate collaboration, lack of guidelines and instigation of a specific investigation into maternal deaths.

**Conclusions:**

This study suggests a number of considerations such as an initiative to collaborate, developing a guideline and presenting training programs before such policies are launched.

## INTRODUCTION

Maternal mortality is defined as death due to either obstetric problems or pre-existing disease which would be exacerbated during pregnancy or 42 days after pregnancy termination.^1^,^2^

Although maternal deaths have reduced significantly over time, from 529,000 to 358000, at 1990 to 2008,^3^ the figure is still high and needs further attention. A significant number of women suffer post-pregnancy morbidity, that is, from disability and health problems.^4^ It is estimated that about 15 % of all pregnant women will face unforeseen difficulties during their pregnancy.^1^

The main targets of the United Nations Millennium Project were defined as decreasing poverty, increasing development, human rights and democracy, and protection of the environment, peace and safekeeping. Maternal and child mortality are indices which are affected by development, poverty and democracy. Therefore improvements in maternal health and reduction of child mortality are indicative of the main goals of the UN Millennium Project achievement.^5^ According to the Millennium Development Goals, maternal mortality ratio should be reduced to %75 by 2015.^6^ Based on the literature, the tragedy of many of maternal deaths in less developed countries is preventable.^7^ Improving the socio-economic situation,^8^ antenatal care,^9^ family planning^10^ and skill attendance^11^ are factors which could lead to reduce the maternal mortality rate.

Therefore, adoption of appropriate strategies to prevent maternal deaths is one of the main plans in many countries including Iran. In order to reduce maternal deaths, Iran has made efforts such as significant improvement in skilled attendance, antenatal care^12^ and family planning.^13^ Acknowledging the importance of making appropriate policies, the usefulness and failure of each new policy is evaluated through different strategies, including use of statistics. Health data are said to be taken note of by policy makers.^14^ They are important in planning, monitoring and evaluating health policies. They are only useful when complete, as incomplete data would distort the true picture. Due to the importance of data on maternal deaths, Iran has adopted a new policy which is explicitly explained in the section on Maternal Mortality and Iran.

## BACKGROUND

### 

#### The Health System in Iran

The health system in Iran comprises a combination of health education and health care.^15^ There is at least one Medical Science University present in each province, which is the official representative of the Ministry of Health and Medical Education in that province.^16^ The District Health Network is the official administrator of health and medical care in the districts. The first level of medical services presentations are the Health Houses in rural areas and the Health Posts in urban areas. These Health Houses are situated in most rural areas (%86), but the growing population of cities prevents the same degree of cover in the urban areas.^17^ The health staff who works at the Rural Health Houses is called Behvarz. The next section explains a brief overview of the efforts made to investigate and control maternal deaths in Iran.

#### Maternal mortality data collection in Iran

In addition to the official source of mortality data, the Civil Registry and the Statistics Unit at the Medical Sciences University, maternal mortality records were collected through the Family Unit at the Medical Sciences University. This strategy was launched by the Maternal and Neonatal Mortality Committee, which was established at 1995 in the national, provincial and local levels in Iran.^18^ The main aim of the committee was the reduction of maternal deaths. In 1996, maternal mortality was assessed through the census; in 1997 a Reproductive Age Mortality Survey (RAMOS) was conducted to estimate the maternal mortality ratio.^18^ The RAMOS questionnaire was reviewed by the Maternal Mortality Committee and a modified version of the RAMOS questionnaire was used to survey the maternal deaths. The results of the new approach assessment at 1999 showed that this system had to be modified for the following reasons; the shortage of complete and accurate data, the lack of classification of maternal deaths based on the International Classification of Cause of Death, and few proper interventions based on the results of the survey. In order to solve these problems the national system of maternal mortality surveillance was set up in 2000.^18^

The next section provides more information about the national system of maternal mortality surveillance.

#### The national maternal mortality surveillance system

In the national maternal mortality surveillance system, there are four main processes present; data collection, investigation of cause of deaths, planning plus intervention, and evaluation. Here is a brief description of each of these ones.

Data collection is conducted via an interview using structured and open-ended questions. Also investigating the recorded documents, and verbal autopsy enrich the information required The collected data would be classified into three groups; verbal autopsy by interviews with relatives of the deceased, private interviews with the health staff involved in provision of health care for the deceased and clinical autopsy.^18^ The next stage is to investigate the causes of mortality in order to find any avoidable ones. It has a crucial role in setting up appropriate intervention and planning.^18^ The committee is responsible for managing the implementation of interventions. Indeed, all details including the intervention, the people who are responsible for carrying it out, and the time of intervention should be clarified by the committee.^18^ The last part of the process is evaluation. The evaluation is undertaken using two basic methods; process evaluation and outcome evaluation. The process evaluation is applied for planning, intervention and evaluation of the intervention. The outcome evaluation is used to appraise the general function of the surveillance system.^18^ The ultimate aim of all mentioned efforts is to reduce maternal mortality ratio.

As detailed above, a clear objective and strategy have been defined at the national level. The question is that whether this plan is able to carry out at the local level. It has been discussed that sometimes an organization functions are contrary to its rules and aims. Therefore it is suggested that these problems can be scrutinized through street level bureaucrats’ behavior and attitudes. Street level bureaucrats were defined by Lipsky as people who interact directly with citizens in the course of their jobs and have substantial discretion in the execution of their work. Health workers are examples of street level bureaucrats.^19^ Based on mentioned explanation, as an independent and external evaluator, this study aimed to explore the gap between policy made at the national level on maternal mortality investigation and the policy implementation at different related levels.

**Table 1:** Comparing the routine works and the Guideline

	The Guideline	The routine
Source of data	hospitals (maternal and nonmaternal), any organization and office reporting maternal deaths data	Urban areas: obstetric wards at the hospitals Rural areas: the Health Houses. No maternal death classification at the Forensic Medicine
reporting	Urgently	reporting with other deaths in rural areas
Evaluation	the process of reporting deaths should be evaluated as process evaluation	No evaluation of the process of reporting of deaths
Definition of maternal death	ICD –9	different descriptions
Likely Source of problems	Inadequate training No guideline availability in different relevant centres risk of conflict : the care delivery are supposed to send the data pointing out a problem	

## METHODS

This qualitative study was a part of a larger study carried out in a town located in Kerman province (Bam), and in Kerman, the centre of Kerman province. Data were collected using in-depth interviews, documents assessment and observation. In order to gain the access to the fieldwork, the permission was obtained from the deputy of the Health Department. Collaboration with Forensic Medicine was performed through a letter issued from Kerman Medical University. To achieve the aim, two groups were interviewed; data collectors who collect and send the data and policy makers who investigate the deaths and had a role in policy design.

Data collectors in rural and urban areas, at the local level, were interviewed to explain the routine process of maternal death reporting. Policy makers at the provincial and local levels were interviewed to discover the problems they perceived in the current system. Additionally, a template was used as a guideline to explain all procedures including data collection and reporting maternal deaths based on the national maternal mortality surveillance system. The template was composed of the documents extracted through the fieldwork interviews. All the interviews were conducted at the Health Workers’ work-place, allowing the direct observation of working practices.

Because of some study limitations such as travel expenses, only some areas were enrolled in the study. Considering that distance from the centre might affect the response; three Rural Health Centres were identified, based on their distance from Bam (outmost, middle, nearest). Regarding the Rural Health House, the most peripheral Health Centre, (each Rural Health Centre covers several Rural Health Houses), one Rural Health House was randomly selected from each of the Rural Health Centres. Therefore, there were three Rural Health Centres and three Rural Health Houses selected in the rural areas.

## RESULTS

The results were categorized in two main parts. The first section highlighted the gap between policy and practice. This was more apparent when comparing the guideline with what was carried out. The second part was composed of findings extracted from the interviews and observations to explain etiologic factors of this gap. Based on the guideline, a considerable number of data sources sending the data to the Family Unit by the Maternal Mortality Committee were identified. These included hospitals, organizations, offices reporting maternal deaths, in women aged between 10 to 49 years to the Statistics Unit and any unofficial persons aware of maternal The guideline suggests reporting these deaths urgently.

However, the results of interviews showed a difference between the guideline suggestions and what was actually carried out The data extracted from the interviews with policy makers indicate that the main data sources in urban and rural areas were obstetric wards at the hospitals and Health Houses respectively. Therefore those maternal deaths occurred with a delay after delivery in the non-obstetrics hospitals/wards could be missed. However any maternal death happens at home needs to be reported to the Forensic Medicine Department in order to issue a death certificate. However, there is no specific classification of maternal deaths present at the Forensic Medicine Department. For example, if a mother dies due to infection following the delivery, the cause of death is infection and is not categorized as a maternal death. The Department was not aware of reporting maternal deaths to the Family Unit exclusively. Further scrutiny in other data sources revealed that some of them were also not aware of this policy. Furthermore, reporting maternal deaths was not perceived as an urgent requirement by one group of respondents, as was suggested by the Committee. In fact, if there is a maternal death, it is reported along with other deaths to the Statistics Unit.

Based on the guideline, the process of deaths reports should be evaluated as process evaluation. However, the interview with policy makers revealed that the main concern of policy makers was the investigation of deaths themselves, not the evaluation of the process death reporting.

The source of this gap was further scrutinized as follows; first: Inadequate training programs which involves two issues; recognizing maternal deaths and reporting them urgently. The Maternal Mortality Committee had adopted the maternal mortality definition from ICD -9, which is defined as a death during pregnancy or 42 days after the delivery. The mother’s age, gestational age, or type of birth were not taken into account as a contributor to maternal mortality.^18^

Different descriptions given by the interviewees and the failure to send the data urgently to the Family Unit, point to the lack of inadequate training schedule for staff before commencing the new policy. Moreover, the only information tool, the guideline, was available in only a limited number of centres which might deal with maternal deaths and the reporting of them.

Another important point which might impede the process of data collection is that following the identification of deaths as maternal ones, these are investigated and if there is a flaw in health care provision, some sort of punishment will be administered.

## DISCUSSION

The discussion should begin with the main findings of the article then the less important results should be mentioned.

The results of this study identified some problems concerning the implementation of the policy announced by the Maternal Mortality Committee. The failure to report maternal deaths from data sources such as the Forensic Medicine Department could be otherwise explained. The Department provides little strategy for better collaboration, poor information systems aimed informing all contributory organs about the new policy, and non-availability of guidelines and competent staff.

Failure to collect complete data could in turn affect the quality of the findings.^20^ The final aim of data collection is transferring the data in order to gain knowledge.^21^ The knowledge is used to take proper action to solve the problems. Both mangers and politicians can use this data to make proper decisions.^22^ In developing countries, although the policy makers tend to make decision based on evidence and know how to use the data, but the quality and availability of data are not satisfactory.^23^ For example, In African Regions the data are not up to date, available, complete and comprehensive.^24^ Hence, it is no surprise that decisions are likely to be made without any evidence. Furthermore, it can affect the usefulness of data when evaluating any intervention policy in the long term. In a study conducted by Macfarlane e et al. in England it was pointed out that lack of data from the private sectors resulted in difficulty in evaluation of the general trends.^25^

Needless to say, if a new policy were to be in place, all relevant parties and stakeholders must be well informed and understand the importance of the new policy. Additionally, the Department’s collaboration can be increased through the sharing of views and concerns.^26^

Another subject to be considered is that even if there were some gains attained by implementing this policy, it would not really be conducted by competent staff. This could be gained through training programs to improve staff knowledge about the significance of the problem. Conditions defined as maternal deaths and the way they are reported should be explained clearly to the relevant staff. Employing technology not only is an effective way of informing people about any changes or new policies, but it can also be used to train staff for target procedures. However, inadequate training of staff procedures is discussed as a significant problem in the work place in developing countries.^27^

Lack of guidelines availability for all related offices is considered as another factor affecting the completeness. Presentation of a proper guideline has many benefits for both data collectors and data users by providing clear goals for data collection and data usage at different levels.^28^

Another issue related with completeness is that due to the instigation of a specific investigation into maternal deaths, the accuracy of data may have deteriorated. Staffs who had the spontaneous responsibilities for patients care and data collection on maternal deaths would report a conflict of interest when targets to reduce maternal mortality were introduced.^14^ There were reports of staff being punished following investigation of a reported maternal death. This could have resulted in non-reporting of maternal deaths or falsification of data. This is of particular concern if decisions relating to health policy and health care resource allocation were to be based on incomplete or inaccurate data. Also, evaluation of data reported by policy makers could be an incentive for staff to report all cases. In contrast, inadequate attention can discourage staff to present the complete set of data.

Finally, the present study might raise this question; do the advantages of implementing this policy weigh up the disadvantages? Establishing a system parallel to the main data collection system may give better quality of data, but it would undermine the main data collection system and the Civil Registry and is detrimental to the entity of the health system. It would also cause duplication, and increase the work load of staff.^29^

This study highlights the problems encountered while gathering maternal deaths data in part of Iran which, in turn, indicates the difficulties of recording a complete set of data. However, as with many other qualitative case studies, generalizing the results to other cities in Iran is of limited validity. Additional studies are recommended in other cities to obtain a general conclusion about the problem. Also the primary aim of this study was a description of maternal deaths reporting system and did not aim to suggest any solution for the problem. Hence studies on possible strategies to tackle the problem are recommended. In addition, exploring maternal mortality ratio trends in the study setting might present a better view of the problem. However, generation of such results goes beyond the remit of this study which is a pure qualitative one.

## CONCLUSION

Although many efforts are intended to prevent maternal deaths, as noted in the results, there are discrepancies between what the committee requires and what is really done at the bottom level of the system. This could question the achievement of primary goals of the Maternal Mortality Committee. Introduction of a new policy requires some preliminaries, such as gathering the opinions of those who will deal with it. Furthermore, it is crucially important to consider the limitations and lack of knowledge at the bottom level to carry out the policy as sometimes an organization might function contrary to its own rules and aims. It should be born in mind that an efficient information system should be used when introducing a new policy and that initiative control criteria are required to ensure that the new policy is understood and carried out. This is of crucial importance to have a complete picture of the story to decrease preventable causes of maternal deaths.
